# Direct δ‐Lactone Synthesis From Free Alcohols via Photoinduced δ‐C(sp^3^)–H Carbonylation in Flow

**DOI:** 10.1002/anie.5570038

**Published:** 2026-05-29

**Authors:** Prakash Chandra Tiwari, Runkang Liu, Timothy Noël

**Affiliations:** ^1^ Flow Chemistry Group Van ’t Hoff Institute for Molecular Sciences (HIMS) University of Amsterdam Amsterdam the Netherlands

**Keywords:** carbonylation, flow chemistry, hydrogen‐atom transfer, photocatalysis, radical chemistry

## Abstract

The direct carbonylation of unactivated C(sp^3^)─H bonds remains a fundamental challenge in synthesis. Herein, we report a photo‐flow strategy for the δ‐C(sp^3^)─H carbonylative lactonization of free alcohols using carbon monoxide. The transformation proceeds via photochemical generation of alkoxyl radicals, selective 1,5‐hydrogen atom transfer, CO trapping to form δ‐acyl radicals, and a crucial radical–polar crossover event that enables intramolecular cyclization to δ‐lactones. The use of a strongly oxidizing acridinium photocatalyst is essential to promote acyl radical oxidation, distinguishing this manifold from conventional proton‐coupled electron transfer (PCET) or ligand‐to‐metal charge transfer (LMCT) pathways. Continuous‐flow conditions facilitate efficient gas–liquid mass transfer and safe handling of CO, enabling short reaction times and scalability. The method tolerates diverse functional groups and provides direct access to structurally complex and bioactive δ‐lactone motifs from simple alcohol feedstocks.

## Graphical abstract

Herein, we have developed a photo‐flow platform for the δ‐C(sp^3^)─H carbonylative lactonization of free alcohols with carbon monoxide. The transformation integrates alkoxyl radical generation, selective 1,5‐HAT, CO incorporation, and radical–polar crossover to enable direct access to δ‐lactones from simple alcohol precursors. The requirement for a high‐potential photooxidant underscores the key role of acyl radical oxidation in enabling productive cyclization. Compared to classical stoichiometric lead‐mediated systems, this strategy proceeds under mild photochemical conditions and benefits from enhanced safety and scalability through continuous‐flow technology. This work establishes a practical and conceptually distinct approach to distal C(sp^3^)─H carbonylation and expands the synthetic utility of free alcohols as radical progenitors.

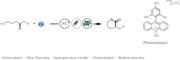

## 1 Introduction

**FIGURE 1:**
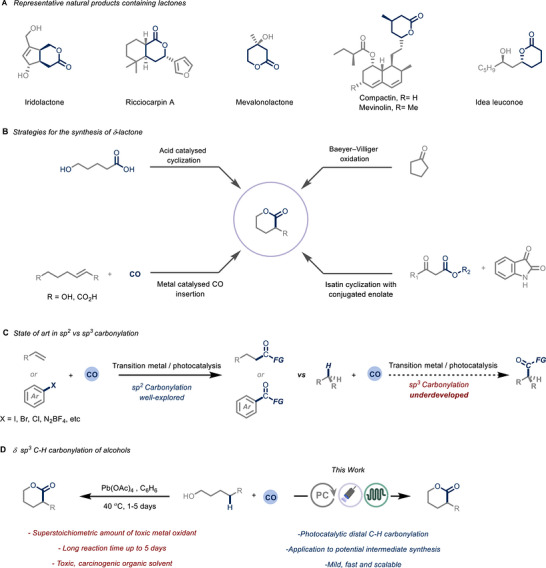
(A) Representative δ‐lactone‐containing natural products. (B) Established strategies for δ‐lactone synthesis. (C) State of the art in carbonylation: C(sp^2^)─X activation and C(sp^2^)─H carbonylation are well developed, whereas site‐selective C(sp^3^)─H carbonylation remains challenging. (D) Distal δ‐C(sp^3^)─H carbonylation of free alcohols: comparison between the Pb(OAc)_4_‐based protocol and the present photo‐flow strategy.

δ‐Lactones are prevalent structural motifs in natural products and bioactive small molecules, and they are frequently used as versatile intermediates in synthesis due to their rich downstream reactivity (Figure 1A) [1, 2, 3, 4, 5]. Consequently, the development of step‐economical and broadly applicable methods for δ‐lactone construction remains an important objective in modern synthetic chemistry (Figure 1B) [6]. Traditional approaches typically access δ‐lactones from prefunctionalized hydroxy acids through intramolecular esterification [7, 8] or by oxidation‐based strategies such as Baeyer–Villiger reactions [9, 10]. While reliable, these methods often require multi‐step preparation of advanced substrates, generate stoichiometric waste, and may suffer from limited functional‐group tolerance, motivating continued interest in more direct and sustainable entry points to δ‐lactones [11, 12, 13].

Carbonylation reactions offer a compelling alternative, as they enable the introduction of carbon monoxide (CO) into organic molecules to rapidly generate carbonyl‐containing products from simple precursors (Figure 1C) [14, 15, 16, 17, 18, 19, 20]. In particular, the carbonylative conversion of readily available alcohols into lactones constitutes an attractive strategy, linking inexpensive feedstocks with high‐value cyclic products. However, despite major progress in transition‐metal‐catalyzed carbonylation chemistry [20, 21], direct carbonylation of unactivated aliphatic C(sp^3^)─H bonds remains challenging due to their high bond dissociation energies, weak innate differentiation, and the difficulty of achieving predictable site‐selectivity [22, 23].

Visible‐light photoredox catalysis has recently emerged as a powerful platform for selective C(sp^3^) ─H functionalization through hydrogen atom transfer (HAT) pathways, including distal reactivity enabled by 1,5‐HAT [24, 25, 26]. Nevertheless, distal δ‐C(sp^3^)─H carbonylation remains strategically underdeveloped, particularly in the context of free alcohols as the radical progenitors [27, 28, 29, 30]. Direct activation of unprotected alcohols is inherently difficult because O─H bonds are exceptionally strong, and many existing 1,5‐HAT manifolds rely on prefunctionalized alkoxy radical precursors (e.g., nitrites, peroxides, or tailored derivatives) [31, 32, 33, 34, 35, 36, 37, 38, 39] rather than simple alcohols. As a consequence, only a limited number of examples are known in which free alcohols undergo δ‐C(sp^3^)─H carbonylation followed by lactonization.

A rare and notable precedent for δ‐lactone synthesis from saturated alcohols and CO was reported using stoichiometric Pb(OAc)_4_ to generate alkoxyl radicals, triggering 1,5‐HAT and subsequent CO trapping (Figure 1D) [40]. Although conceptually powerful, this oxidative system requires super‐stoichiometric amounts of a toxic lead reagent, employs benzene as solvent, and typically proceeds over several days, imposing significant practical and sustainability limitations. More broadly, these constraints illustrate the key unmet need: a general, mild, and operationally safe platform for δ‐C(sp^3^)─H carbonylation of simple alcohols that can deliver δ‐lactones efficiently and with practical reaction times [41, 42, 43].

Herein, we report a photo‐flow strategy for the distal δ‐C(sp^3^)─H carbonylative lactonization of free alcohols using carbon monoxide (Figure 1D). Our approach integrates photochemical alkoxyl‐radical generation and 1,5‐HAT to access δ‐carbon‐centered radicals, followed by CO incorporation to form acyl radical intermediates. Crucially, productive δ‐lactone formation is enabled by a photocatalytic radical–polar crossover (RPC) step, converting acyl radicals into acylium‐type intermediates that undergo rapid intramolecular cyclization. In addition, the use of continuous‐flow technology provides a controlled and scalable means to handle pressurized CO, enhancing gas–liquid mass transfer and enabling short residence times while maintaining operational safety [44, 45, 46, 47, 48]. This methodology enables the direct synthesis of δ‐lactones from readily available alcohol feedstocks across a broad substrate scope, including functionalized and cyclic architectures, thus offering a practical and conceptually distinct alternative to classical stoichiometric lead‐mediated protocols.

**FIGURE 2:**
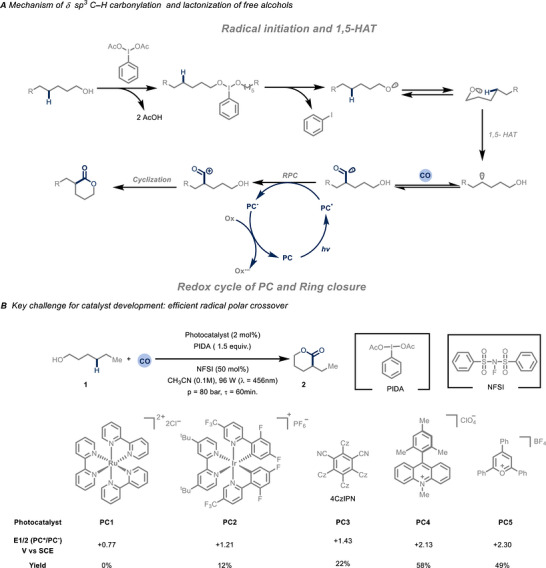
(A) Mechanistic proposal for the carbonylative δ‐C(sp^3^)─H lactonization of free alcohols. (B) Catalyst development: effect of the photocatalyst on single‐electron oxidation. Reactions performed on a 0.2 mmol scale, 96 W of 456‐nm LEDs. ^a)^Yields determined by ^1^H NMR using trichloroethylene as an external standard. ^b)^See Supporting Information for experimental details.

We began our investigation by outlining a mechanistic blueprint for the carbonylative δ‐lactonization of free alcohols (Figure 2A). The transformation was envisioned to proceed through initial generation of an oxygen‐centered radical, followed by a selective 1,5‐hydrogen atom transfer (1,5‐HAT) to form a δ‐C(sp^3^)‐centered radical. Trapping of this carbon‐centered radical with carbon monoxide would furnish a δ‐acyl radical intermediate. Crucially, productive lactone formation would require oxidation of this acyl radical via a RPC event to generate an acylium‐type intermediate, which could undergo intramolecular nucleophilic attack by the pendant alcohol and subsequent deprotonation to afford the desired δ‐lactone.

Thus, a central design element of this strategy is the selective one‐electron oxidation of the acyl radical intermediate. While proton‐coupled electron transfer (PCET) and ligand‐to‐metal charge transfer (LMCT) strategies have emerged as powerful approaches for X─H bond activation and O‐centered radical generation, these manifolds typically operate through reductive quenching of the photocatalyst or metal center [28, 29, 30]. As a consequence, they do not readily enable a subsequent oxidative radical–polar crossover step within the same catalytic cycle. Consistent with this mechanistic constraint, no product formation was observed under representative PCET or LMCT conditions (see Supporting Information).

Recent studies have demonstrated that hypervalent iodine reagents can promote 1,5‐HAT processes through photochemical generation of alkoxyl radicals [49, 50]. We therefore hypothesized that combining hypervalent iodine‐mediated *O*‐radical formation with a sufficiently strong photooxidant could provide a viable platform for *δ*‐C(sp^3^)─H carbonylation followed by RPC‐enabled lactonization.

To evaluate this concept, we selected the carbonylative conversion of commercially available hexane‐1‐ol (**1**) to δ‐lactone (**2**) under continuous‐flow conditions as a model reaction. Initial experiments employing [Ru(bpy)_3_]Cl_2_ [*E*_1_/_2_(PC*/PC) = +0.77] or [Ir[dF(CF_3_)_2_ppy]_2_(bpy)]PF_6_ [*E*_1_/_2_(PC*/PC) = +1.21] proved ineffective, affording at most 12% yield of the desired product (Figure 2B). We attributed this low efficiency to insufficient oxidizing power to promote the key radical–polar crossover step.

Given the high excited‐state oxidation potentials of acridinium‐based photocatalysts, we next evaluated **Mes‐Acr‐Me** ^+^ [*E*1/2(PC^*^/PC) = +2.13] as a photooxidant. Upon visible‐light irradiation, the excited‐state *Mes‐Acr‐Me^+^ can oxidize the acyl radical intermediate, generating an acylium‐type species and the reduced **Mes‐Acr‐Me•** radical. The latter is subsequently reoxidized by the stoichiometric oxidant present in the reaction mixture, thereby closing the catalytic cycle.

Under optimized flow conditions, merging a 0.1 M acetonitrile solution of hexane‐1‐ol (**1**) containing 2 mol% **PC4**, 1.5 equiv of (diacetoxyiodo)benzene (PIDA), and 50 mol% *N*‐fluorobenzenesulfonimide (NFSI) with a stream of CO and irradiating at 456 nm afforded δ‐lactone (**2**) in 58% ^1^H NMR yield (Table 1, entry 1). Extending the residence time to 90 min led to diminished yield (45%, entry 2), likely due to product decomposition under prolonged irradiation [51]. Increasing the loading of either **PC4** or PIDA did not improve the outcome (entries 3–4), suggesting that overoxidation or competitive side reactions may become operative at higher oxidant concentrations. Variation of the gas‐to‐liquid ratio (G:L) from 40:1 to 60:1 (v/v) resulted in a diminished yield of **2** (48%, Table 1, entry 5), indicating that the quantity of CO supplied is not a limiting factor for acyl radical trapping. The decrease in yield can be likely attributed to changes in hydrodynamics at higher gas fractions and the reduced liquid throughput, potentially affecting photon flux and mixing efficiency within the microreactor [52]. A more pronounced decrease in efficiency was observed upon lowering the pressure to 40 bar, affording only 37% yield (entry 6). This trend is consistent with Henry's law, as the solubility of carbon monoxide in solution is directly proportional to its partial pressure; reduced CO concentration therefore limits productive acyl radical trapping. Control experiments further confirmed the necessity of each reaction component, as omission of the photocatalyst, oxidant, light, or NFSI resulted in either complete inhibition or substantially diminished lactone formation (entries 7–10; see Supporting Information for details).

**TABLE 1:** Optimization of the lactonization of 1 with CO.

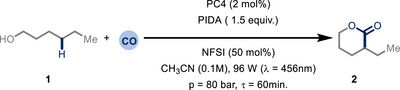		

Entry	Deviation	Yield of 2^a^
1	None	58%
2	*t*_R_ = 90 min	45%
3	PC4 (5 mol%)	51%
4	PIDA (2.0 equiv)	52%
5	60:1 (G: L)	48%
6	p = 40 bar	37%
7	No NFSI	49%
8	No light	n.d.^b^
9	No PC4	n.d.^b^
10	No PIDA	n.d.^b^

Reactions performed on a 0.2 mmol scale, 96 W of 456‐nm LEDs.

^a^ Yields determined by ^1^H NMR using trichloroethylene as an external standard.

^b^ See Supporting Information for experimental details.

**FIGURE 3:**
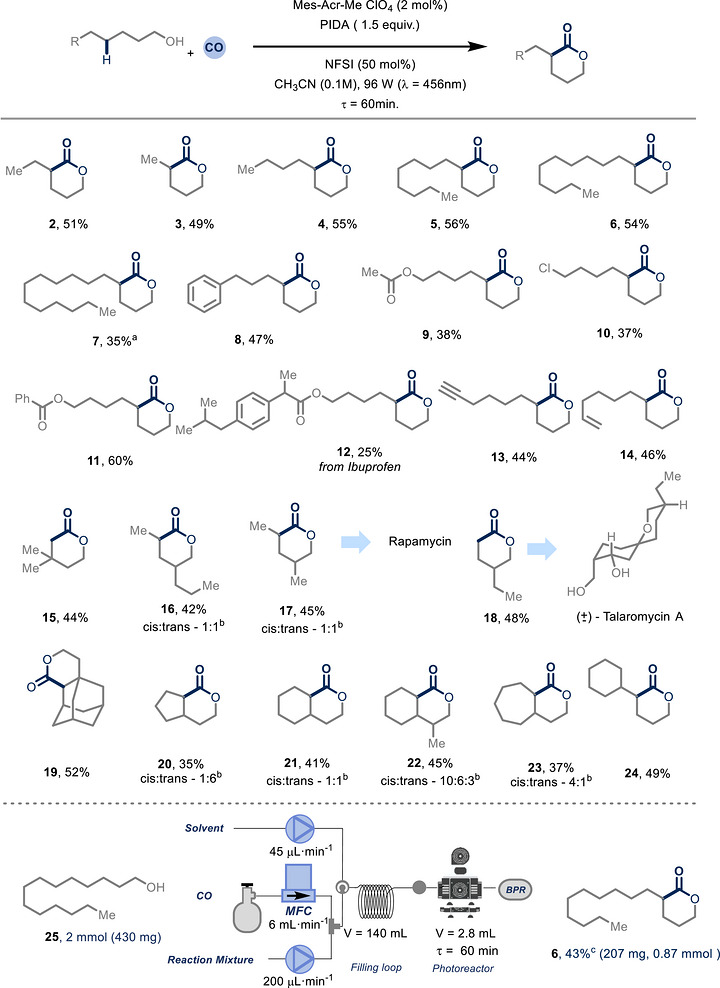
Scope of the lactonization of free alcohols with CO. Reaction conditions: alcohol (0.3 mmol. 1.0 equiv), PC4 (2 mol%), PIDA (1.5 equiv), and NFSI (50 mol%) in 3 mL of CH_3_CN, G:L = 40:1, 96 W of 456 nm LEDs. All yields are those of isolated products (See Supporting Information for experimental details). ^a)^CH_3_CN: Pentane (4:1) were used as a solvent. ^b)^Cis/Trans ratio was determined by ^1^H NMR or GC‐FID. ^c)^Scale‐up conditions: alcohol (2.0 mmol. 1.0 equiv), PC4 (2 mol %), PIDA (1.5 equiv), and NFSI (50 mol%) in 20 mL of CH_3_CN (0.1 M), G:L = 30:1, 96 W of 456 nm LEDs (See Supporting Information for experimental details).

Under these optimized conditions, we evaluated the substrate scope of this method for lactone synthesis (Figure 3A). We initially focused on aliphatic alcohols with varying chain lengths (C5─C16), and the lactonization proceeded smoothly, affording good yields (**2‐7**). Alcohols containing functional groups such as phenyl, halogen, and acetyl produced the desired lactones in moderate to good yields (**8‐11**). Of particular note, Ibuprofen ester analogues of alcohol, afforded the desired product in moderate yield (**12**). Functional groups such as alkynes, as shown in entry **13**, remained unaffected. The developed catalytic system did not oxidize monosubstituted alkenes, allowing the selective formation of a lactone from an alkene‐containing alcohol **14**.

In the modern era of synthesis, achieving step and atom economical syntheses of bioactive compounds is a major driver. With this in mind, we focused on alcohols bearing substitutions at the β and γ positions, which serve as key intermediates in the synthesis of numerous natural products. An alcohol bearing a 3‐methyl group at the γ position was smoothly converted into the corresponding lactone (**15**). Similarly, a β, δ‐disubstituted lactone (**16**) was obtained from an alcohol containing a 2‐propyl group at the β position. Lactone 3,5‐dimethyltetrahydro‐2H‐pyran‐2‐one which is a key intermediate in the synthesis of Rapamycin natural product was successfully synthesized in 45% yield (**17**). Interestingly, the racemic lactone 5‐ethyltetrahydro‐2H‐pyran‐2‐one, which has been used in the total synthesis of racemic Talaromycin A, was prepared from the commercially available alcohol in a single step (**18**).

To further showcase the synthetic utility of this protocol, we turned our attention to the synthesis of bi‐ and polycyclic lactones. Under our optimized conditions, a polycyclic lactone was readily synthesized from 1‐adamantaneethanol in 52% isolated yield (**19**). Similarly, bicyclic lactones with varying ring sizes, including 6,6‐fused, 5,6‐fused, and 7,6‐fused systems, were synthesized in synthetically useful yields (**20–23**).

Considering the use of CO and potential application of this reaction on a large scale, we tested a 2‐mmol‐scale reaction with (**25**) using 2 mol % photocatalyst loading and 30:1 G:L (Figure 3) allowing safe, controlled, and scalable use of CO. Chromatographic purification gave lactone product **6** in 43% yield. The 0.3‐mmol‐scale gave a 54% yield of **6**, under slightly modified conditions demonstrating the scalability of this method.

In summary, we have developed a photo‐flow platform for the δ‐C(sp^3^)─H carbonylative lactonization of free alcohols with carbon monoxide. The transformation integrates alkoxyl radical generation, selective 1,5‐HAT, CO incorporation, and RPC to enable direct access to δ‐lactones from simple alcohol precursors. The requirement for a high‐potential photooxidant underscores the key role of acyl radical oxidation in enabling productive cyclization. Compared to classical stoichiometric lead‐mediated systems, this strategy proceeds under mild photochemical conditions and benefits from enhanced safety and scalability through continuous‐flow technology. This work establishes a practical and conceptually distinct approach to distal C(sp^3^)─H carbonylation and expands the synthetic utility of free alcohols as radical progenitors.

## Author Contributions

**Prakash Chandra Tiwari**: conceptualization, investigation, writing – original draft, methodology, formal analysis, data curation. **Runkang Liu**: Investigation, methodology, formal analysis. **Timothy Noël**: conceptualization, writing – review and editing, supervision, project administration, funding acquisition.

## Conflicts of Interest

The authors declare no conflicts of interest.

## Supporting information




**Supporting File**: anie72897‐sup‐0001‐SuppMat.docx.
The authors have cited additional references within the Supporting Information [1, 2, 3, 4, 5, 6].

## Data Availability

The data that supports the findings of this study are available in the Supporting Information of this article.

## References

[1] J. Rocca , J. Tumlinson , B. Glancey , and C. Lofgren , “The Queen Recognition Pheromone of , Preparation of (‐6‐(1‐Pentenyl)‐2H‐Pyran‐2‐One,” Tetrahedron Letters 24 (1983): 1889–1892, 10.1016/S0040-4039(00)81798-0.

[2] A. Endo , M. Kuroda , and Y. Tsujita , “ML‐236A, ML‐236B, and ML‐236C, New Inhibitors of Cholesterogensis Produced by Penicillium Citrinum,” Journal of Antibiotics (Tokyo) 29 (1976): 1346–1348, 10.7164/antibiotics.29.1346. 1010803

[3] K. Mori , “Synthesis of Optically Active Pheromones,” Tetrahedron 45 (1989): 3233–3298, 10.1016/S0040-4020(01)81007-3.

[4] G. J. Florence , N. M. Gardner , and I. Paterson , “Development of Practical Syntheses of the Marine Anticancer Agents Discodermolide and Dictyostatin,” Natural Product Reports 25 (2008): 342, 10.1039/b705661n. 18389141

[5] P. Chiu , L. T. Leung , and B. C. B. Ko , “Pseudolaric Acids: Isolation, Bioactivity and Synthetic Studies,” Natural Product Reports 27 (2010): 1066, 10.1039/b906520m. 20405078

[6] K. Palanichamy and K. P. Kaliappan , “Synthesis of Saturated Six‐Membered Ring Lactones,” Synthesis of Saturated Oxygenated Heterocycles I. Topics in Heterocyclic Chemistry In: J. Cossy , eds. 35 (Springer, 2014): 97–140, 10.1007/978-3-642-41473-2_3

[7] R. E. Counsell , P. D. Klimstra , and F. B. Colton , “Anabolic Agents. Derivatives of 5α‐Androst‐1‐ene,” Journal of Organic Chemistry 27 (1962): 248–253, 10.1021/jo01048a060. 14056384

[8] S. K. Kang , S. G. Kim , D. C. Park , J. S. Lee , W. J. Yoo , and C. S. Pak , “Reductive Elimination of Acetonides, Cyclic Carbonates, or Cyclic Sulfites of γ,δ‐Dihydroxy (E)‐α,β‐Unsaturated Esters: An Efficient Route to δ‐Hydroxy (E)‐β,γ‐Unsaturated Esters and δ‐Hydroxy Esters,” Journal of the Chemical Society, Perkin Transactions 1 (1993): 9–10, 10.1039/P19930000009.

[9] S. Hacini and M. Santelli , “An Efficient Synthesis of the Prelog‐Djerassi Lactone Methyl Ester From (‐)‐Trans‐Pulegenic Acid,” Tetrahedron 46 (1990): 7787–7792, 10.1016/S0040-4020(01)90075-4.

[10] B. Adger , M. T. Bes , G. Grogan , et al., “Application of Enzymic Baeyer–Villiger Oxidations of 2‐substituted Cycloalkanones to the Total Synthesis of (R)‐(+)‐Lipoic Acid,” Journal of the Chemical Society, Chemical Communications (1995): 1563–1564, 10.1039/C39950001563.

[11] J. Yang , B. W. Pan , L. Chen , Y. Zhou , and X. L. Liu , “Recent Advances in Organocatalytic Cascade Reactions for Enantioselective Synthesis of Chiral Spirolactone Skeletons,” Chem Synth 3 (2023): 7, 10.20517/cs.2022.38.

[12] C. U. Grünanger and B. Breit , “Remote Control of Regio‐ and Diastereoselectivity in the Hydroformylation of Bishomoallylic Alcohols With Catalytic Amounts of a Reversibly Bound Directing Group,” Angewandte Chemie International Edition 49 (2010): 967–970, 10.1002/anie.200905949. 20029868

[13] U. Annby , M. Stenkula , and C. M. Andersson , “Regiochemistry of Palladium(II)‐Assisted Oxidative Lactonisation Reactions,” Tetrahedron Letters 34 (1993): 8545–8548, 10.1016/S0040-4039(00)61381-3.

[14] M. Beller and X. F. Wu , Transition Metal Catalyzed Carbonylation Reactions (Springer, 2013). 10.1021/ar400222k 24564478

[15] J. B. Peng , F. P. Wu , and X. F. Wu , “First‐Row Transition‐Metal‐Catalyzed Carbonylative Transformations of Carbon Electrophiles,” Chemical Reviews 119 (2019): 2090–2127, 10.1021/acs.chemrev.8b00068. 29722527

[16] M. L. Clarke , “Hydroformylation. Fundamentals, Processes, and Applications in Organic Synthesis. By Armin Börner and Robert Franke,” Angewandte Chemie International Edition 55 (2016): 13377–13377, 10.1002/anie.201607967.

[17] J. H. A. Schuurmans , T. M. Masson , S. D. A. Zondag , et al., “Light‐Assisted Carbon Dioxide Reduction in an Automated Photoreactor System Coupled to Carbonylation Chemistry,” Chemical Science 15 (2024): 19842–19850, 10.1039/D4SC06660J. 39568953 PMC11575595

[18] Z. P. Bao , L. C. Wang , and X. F. Wu , “Carbonylation: Unlocking Opportunities for Bioactive Molecule and Pharmaceutical Development,” ACS Catalysis 15 (2025): 19580–19606, 10.1021/acscatal.5c07031.

[19] C. S. Kuai , Y. Yuan , and X. F. Wu , “Emerging Trends in CO Carbonylation,” Chemistry 11 (2025): 102503, 10.1016/j.chempr.2025.102503.

[20] T. Kawamoto , T. Fukuyama , B. Picard , and I. Ryu , “New Directions in Radical Carbonylation Chemistry: Combination With Electron Catalysis, Photocatalysis and Ring‐Opening,” Chemical Communications 58 (2022): 7608–7617, 10.1039/D2CC02700C. 35758516

[21] M. Majek and A. Jacobi Von Wangelin , “Metal‐Free Carbonylations by Photoredox Catalysis,” Angewandte Chemie International Edition 54 (2015): 2270–2274, 10.1002/anie.201408516. 25414135

[22] D. Willcox , B. G. N. Chappell , K. F. Hogg , J. Calleja , A. P. Smalley , and M. J. Gaunt , “A General Catalytic β‐C–H Carbonylation of Aliphatic Amines to β‐Lactams,” Science 354 (2016): 851–857, 10.1126/science.aaf9621. 27856900

[23] F. Raymenants , T. M. Masson , J. Sanjosé‐Orduna , and T. Noël , “Efficient C(sp^3^)−H Carbonylation of Light and Heavy Hydrocarbons With Carbon Monoxide via Hydrogen Atom Transfer Photocatalysis in Flow**,” Angewandte Chemie International Edition 62 (2023): e202308563, 10.1002/anie.202308563. 37459232

[24] W. Guo , Q. Wang , and J. Zhu , “Visible Light Photoredox‐Catalysed Remote C–H Functionalisation Enabled by 1,5‐Hydrogen Atom Transfer (1,5‐HAT),” Chemical Society Reviews 50 (2021): 7359–7377, 10.1039/D0CS00774A. 34013927

[25] G. J. Choi , Q. Zhu , D. C. Miller , C. J. Gu , and R. R. Knowles , “Catalytic Alkylation of Remote C–H Bonds Enabled by Proton‐Coupled Electron Transfer,” Nature 539 (2016): 268–271, 10.1038/nature19811. 27732585 PMC5704892

[26] J. C. K. Chu and T. Rovis , “Amide‐Directed Photoredox‐Catalysed C–C Bond Formation at Unactivated sp^3^ C–H Bonds,” Nature 539 (2016): 272–275, 10.1038/nature19810. 27732580 PMC5574171

[27] X. Wu , M. Wang , L. Huan , D. Wang , J. Wang , and C. Zhu , “Tertiary‐Alcohol‐Directed Functionalization of Remote C(sp^3^)−H Bonds by Sequential Hydrogen Atom and Heteroaryl Migrations,” Angewandte Chemie International Edition 57 (2018): 1640–1644, 10.1002/anie.201709025. 29276816

[28] A. Hu , J. J. Guo , H. Pan , H. Tang , Z. Gao , and Z. Zuo , “δ‐Selective Functionalization of Alkanols Enabled by Visible‐Light‐Induced Ligand‐to‐Metal Charge Transfer,” Journal of the American Chemical Society 140 (2018): 1612–1616, 10.1021/jacs.7b13131. 29381061

[29] L. Huang , T. Ji , and M. Rueping , “Remote Nickel‐Catalyzed Cross‐Coupling Arylation via Proton‐Coupled Electron Transfer‐Enabled C–C Bond Cleavage,” Journal of the American Chemical Society 142 (2020): 3532–3539, 10.1021/jacs.9b12490. 32017543

[30] E. Tsui , H. Wang , and R. R. Knowles , “Catalytic Generation of Alkoxy Radicals From Unfunctionalized Alcohols,” Chemical Science 11 (2020): 11124–11141, 10.1039/D0SC04542J. 33384861 PMC7747465

[31] J. Robertson , J. Pillai , and R. K. Lush , “Radical Translocation Reactions in Synthesis,” Chemical Society Reviews 30 (2001): 94–103, 10.1039/b000705f.

[32] K. Chen , J. M. Richter , and P. S. Baran , “1,3‐Diol Synthesis via Controlled, Radical‐Mediated C−H Functionalization,” Journal of the American Chemical Society 130 (2008): 7247–7249, 10.1021/ja802491q. 18481847

[33] Y. Shi , B. Yang , S. Cai , and S. Gao , “Total Synthesis of Gracilamine,” Angewandte Chemie International Edition 53 (2014): 9539–9543, 10.1002/anie.201405418. 25044967

[34] Q. Qin and S. Yu , “Visible‐Light‐Promoted Remote C(sp ^3^)–H Amidation and Chlorination,” Organic Letters 17 (2015): 1894–1897, 10.1021/acs.orglett.5b00582. 25853884

[35] C. Martínez and K. Muñiz , “An Iodine‐Catalyzed Hofmann–Löffler Reaction,” Angewandte Chemie International Edition 54 (2015): 8287–8291, 10.1002/anie.201501122. 26016458

[36] J. Zhang , Y. Li , F. Zhang , C. Hu , and Y. Chen , “Generation of Alkoxyl Radicals by Photoredox Catalysis Enables Selective C(sp^3^)−H Functionalization Under Mild Reaction Conditions,” Angewandte Chemie International Edition 55 (2016): 1872–1875, 10.1002/anie.201510014. 26680274

[37] E. A. Wappes , S. C. Fosu , T. C. Chopko , and D. A. Nagib , “Triiodide‐Mediated δ‐Amination of Secondary C−H Bonds,” Angewandte Chemie International Edition 55 (2016): 9974–9978, 10.1002/anie.201604704. 27384522 PMC5166987

[38] P. Becker , T. Duhamel , C. J. Stein , M. Reiher , and K. Muñiz , “Cooperative Light‐Activated Iodine and Photoredox Catalysis for the Amination of C−H Bonds,” Angewandte Chemie International Edition 56 (2017): 8004–8008, 10.1002/anie.201703611. 28488354 PMC5499658

[39] L. Chang , Q. An , L. Duan , K. Feng , and Z. Zuo , “Alkoxy Radicals See the Light: New Paradigms of Photochemical Synthesis,” Chemical Reviews 122 (2022): 2429–2486, 10.1021/acs.chemrev.1c00256. 34613698

[40] S. Tsunoi , I. Ryu , T. Okuda , M. Tanaka , M. Komatsu , and N. Sonoda , “New Strategies in Carbonylation Chemistry: The Synthesis of δ‐Lactones From Saturated Alcohols and CO,” Journal of the American Chemical Society 120 (1998): 8692–8701, 10.1021/ja9807892.

[41] A. A. H. Laporte , T. M. Masson , S. D. A. Zondag , and T. Noël , “Multiphasic Continuous‐Flow Reactors for Handling Gaseous Reagents in Organic Synthesis: Enhancing Efficiency and Safety in Chemical Processes,” Angewandte Chemie International Edition 63 (2024): e202316108, 10.1002/anie.202316108. 38095968

[42] L. Capaldo , Z. Wen , and T. Noël , “A Field Guide to Flow Chemistry for Synthetic Organic Chemists,” Chemical Science 14 (2023): 4230–4247, 10.1039/D3SC00992K. 37123197 PMC10132167

[43] S. Bonciolini , A. Pulcinella , and T. Noël , “Tech‐Enhanced Synthesis: Exploring the Synergy Between Organic Chemistry and Technology,” Journal of the American Chemical Society 147 (2025): 28523–28545, 10.1021/jacs.5c10303. 40762023 PMC12356545

[44] G. Laudadio , Y. Deng , K. van der Wal , et al., “C(sp^3^)–H Functionalizations of Light Hydrocarbons Using Decatungstate Photocatalysis in Flow,” Science 369 (2020): 92–96, 10.1126/science.abb4688. 32631892

[45] P. C. Tiwari , A. Pulcinella , E. Hodžić , and T. Noël , “Late‐Stage Heteroarene Alkylation via Minisci Reaction With Gaseous Alkanes Enabled by Hydrogen Atom Transfer in Flow,” ACS Central Science 11 (2025): 910–917, 10.1021/acscentsci.5c00468. 40585795 PMC12203263

[46] A. Pulcinella , P. Chandra Tiwari , A. Luridiana , et al., “C1‐4 Alkylation of Aryl Bromides With Light Alkanes Enabled by Metallaphotocatalysis in Flow,” Angewandte Chemie International Edition 64 (2025): e202413846, 10.1002/anie.202413846. 39192732 PMC11720381

[47] L. Buglioni , F. Raymenants , A. Slattery , S. D. A. Zondag , and T. Noël , “Technological Innovations in Photochemistry for Organic Synthesis: Flow Chemistry, High‐Throughput Experimentation, Scale‐up, and Photoelectrochemistry,” Chemical Reviews 122 (2022): 2752–2906, 10.1021/acs.chemrev.1c00332. 34375082 PMC8796205

[48] D. Nagornîi , F. Raymenants , N. Kaplaneris , and T. Noël , “C(sp^3^)–H Sulfinylation of Light Hydrocarbons With Sulfur Dioxide via Hydrogen Atom Transfer Photocatalysis in Flow,” Nature Communications 15 (2024): 5246, 10.1038/s41467-024-49322-w. PMC11186823 38897988

[49] X. Wu , H. Zhang , N. Tang , et al., “Metal‐Free Alcohol‐Directed Regioselective Heteroarylation of Remote Unactivated C(sp^3^)–H Bonds,” Nature Communications 9 (2018): 3343, 10.1038/s41467-018-05522-9. PMC6104081 30131495

[50] G. X. Li , X. Hu , G. He , and G. Chen , “Photoredox‐Mediated Remote C(sp^3^)–H Heteroarylation of Free Alcohols,” Chemical Science 10 (2019): 688–693, 10.1039/C8SC04134B. 30774869 PMC6345347

[51] J. H. A. Schuurmans , F. Lukas , P. C. Tiwari , and T. Noël , “Photon Management in Photochemical Synthesis and Reactor Scale‐Up,” Accounts of Chemical Research 59 (2026): 788–801, 10.1021/acs.accounts.5c00885. 41712308

[52] J. H. A. Schuurmans , S. D. A. Zondag , A. Chaudhuri , M. Claros , J. van der Schaaf , and T. Noël , “Interaction of Light With Gas–Liquid Interfaces: Influence on Photon Absorption in Continuous‐Flow Photoreactors,” Reaction Chemistry & Engineering 10 (2025): 790–799, 10.1039/D4RE00540F. 39816783 PMC11726180

