# Supplementary material for: Direct δ‐Lactone Synthesis From Free Alcohols via Photoinduced δ‐C(sp3)–H Carbonylation in Flow
Source: Angew Chem Int Ed Engl. 2026 May 29;65(29):e5570038. doi: 10.1002/anie.5570038 (PMC13360553; doi:10.1002/anie.5570038)
Supplement: Supplementary file 1 — Supporting File: anie72897‐sup‐0001‐SuppMat.docx. The authors have cited additional references within the Supporting Information [1, 2, 3, 4, 5, 6]. [file ANIE-65-e5570038-s001.docx]

**Direct δ-Lactone Synthesis from Free Alcohols via Photoinduced δ-C(sp³)–H Carbonylation in Flow**

Supplementary Information

*Prakash Chandra Tiwari^1^, Runkang Liu^1^, Timothy Noël^1,^**

^1^Flow Chemistry Group, Van ’t Hoff Institute for Molecular Sciences (HIMS), University of Amsterdam, Science Park 904, 1098 XH Amsterdam, The Netherlands.

** Email:* [*t.noel@uva.nl*](mailto:t.noel@uva.nl) *(T. Noel)*

**Keywords:** Carbonylation • Flow chemistry • Hydrogen-atom transfer • Photocatalysis • Radical chemistry

This PDF file includes:

Materials and Methods

Figures S1 to S5

Tables S1 to S9

References

NMR Data

Table of contents

[1. General information 3](#_Toc229389383)

[2. Reactor Design 4](#_Toc229389384)

[2.1 Flow Equipment 4](#_Toc229389385)

[2.2 Eagle Reactor 5](#_Toc229389386)

[3. General Procedure for the Optimization of gas-liquid Reactions in Flow 6](#_Toc229389387)

[4. Reaction Optimization 7](#_Toc229389388)

[4.1 Photocatalyst screening 7](#_Toc229389389)

[4.2 Photocatalyst amount screening 8](#_Toc229389390)

[4.3 Residence time screening 9](#_Toc229389391)

[4.4 Pressure screening 9](#_Toc229389392)

[4.5 Hypervalent iodine source screening 10](#_Toc229389393)

[4.6 PIDA amount screening 10](#_Toc229389394)

[4.7 Additive screening 11](#_Toc229389395)

[4.8 NFSI amount screening 12](#_Toc229389396)

[4.9 Light intensity screening 12](#_Toc229389397)

[4.10 Exploration of LMCT Photocatalysis 13](#_Toc229389398)

[5. General Procedures (GP) 13](#_Toc229389399)

[5.1 (GP1) 13](#_Toc229389400)

[5.2 Scale-up 13](#_Toc229389401)

[6. Characterization data of synthesized compound 15](#_Toc229389402)

[7. Mechanistic Investigation: 22](#_Toc229389403)

[7.1 Radical trapping with TEMPO: 22](#_Toc229389404)

[7.2 Attempt to trap the acyl cation: 23](#_Toc229389405)

[8. References 23](#_Toc229389406)

[9. NMR Spectra 25](#_Toc229389407)

# General information

**Materials.** All reagents and solvents were used as received without further purification. Reagents and solvents were bought from Sigma Aldrich, TCI and Fluorochem. Technical solvents were bought from VWR International and used as received. N-butane gas with 3.5purity was purchased from Praxair, propane gas with 2.5purity was purchased from Benegas, ethane gas with 3.5purity was purchased from Gerling and Holz and Co and methane gas with 4.5purity was purchased from Nippon gases. Disposable syringes were purchased from Laboratory Glass Specialist. Syringe pumps were purchased from Chemix Inc. model Fusion 200 Touch. All capillary tubing, microfluidic fittings and Back Pressure Regulator (BPR) were purchased from IDEX Health & Science. Product isolation was performed automatically, by a Biotage® Isolation Four, with Biotage® SNAP KP-Sil 4 or 10 g flash chromatography cartridges, or manually, using silica (P60, SILICYCLE). TLC analysis was performed using Silica on aluminum foils TLC plates (F254, SILICYCLE) with visualization under ultraviolet light (254 nm and 365 nm) or appropriate TLC staining (potassium permanganate). Organic solutions were concentrated under reduced pressure on a Büchi rotary evaporator (in vacuo at 40 ºC, ~5 mbar). The synthesis of starting materials 8-hydroxyoctyl acetate^1^, 8-hydroxyoctyl benzoate and 8-hydroxyoctyl 2-(4-isobutylphenyl) propanoate^1^ were prepared following literature procedures.

**NMR spectroscopy.** ^1^H (400 and 300 MHz), ^13^C (101 and 128 MHz), ^19^F (282 and 376 MHz) spectra were recorded at ambient temperature using Bruker AV 300-I, AV 400 and AV 500-NEO. ^1^H NMR spectra are reported in parts per million (ppm) downfield relative to CDCl_3_ (7.26 ppm) and all ^13^C NMR spectra are reported in ppm relative to CDCl_3_ (77.16 ppm) unless stated otherwise. The multiplicities of signals are designated by the following abbreviations: s (singlet), d (doublet), t (triplet), q (quartet), p (pentet), sext (sextet), m (multiplet), dd (doublet of doublets), dt (doublet of triplets), td (triplet of doublets), ddd (doublet of doublet of doublets). Coupling constants (J) are reported in hertz (Hz). NMR data was processed using the MestReNova 14 software package. Known products were characterized by comparing to the corresponding ^1^HNMR, ^13^C NMR, ^19^F NMR with those available in the literature.

**Mass spectrometry.** High resolution mass spectra (HRMS) were collected on an AccuTOF GC v 4g, JMS-T100GCV Mass spectrometer (JEOL, Japan).

**Determination of Cis/Trans Ratio.** All the reported cis/trans ratios referred to the isolated products after column chromatography.

# Reactor Design

## Flow Equipment


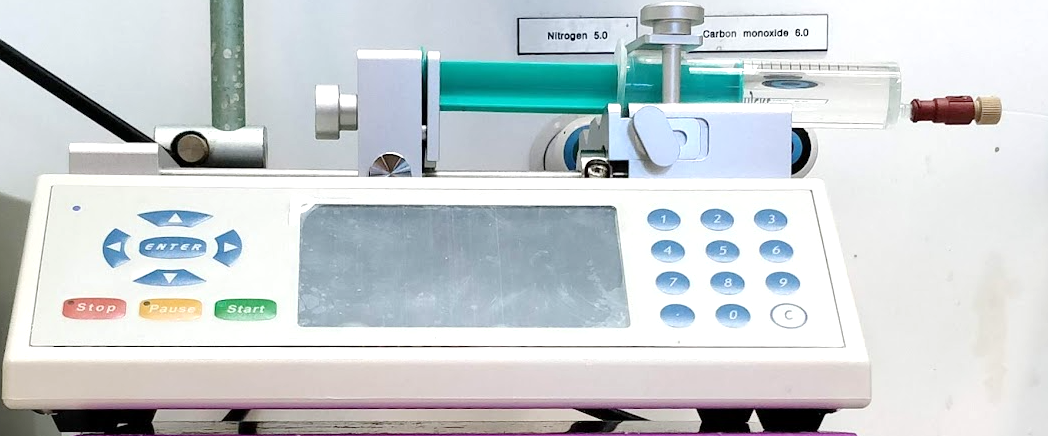


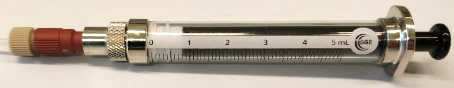


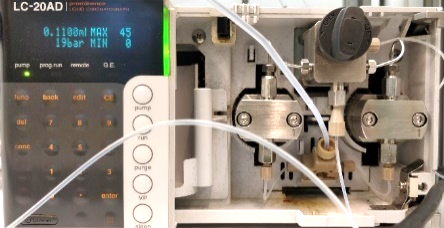


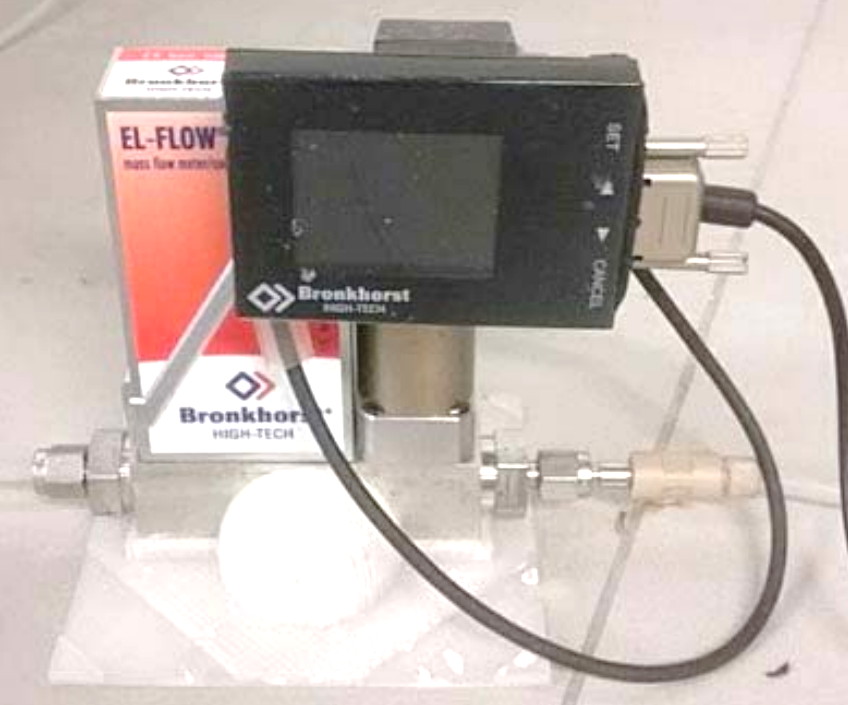


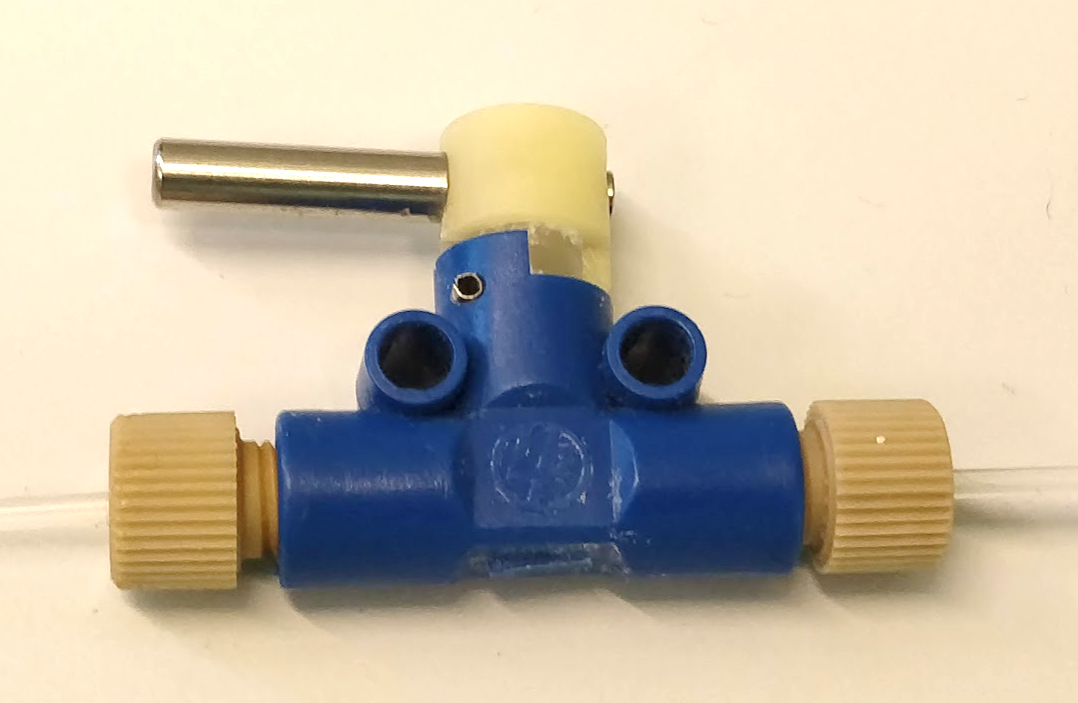


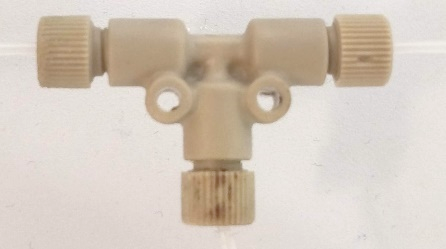


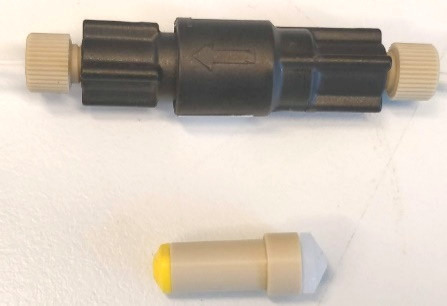


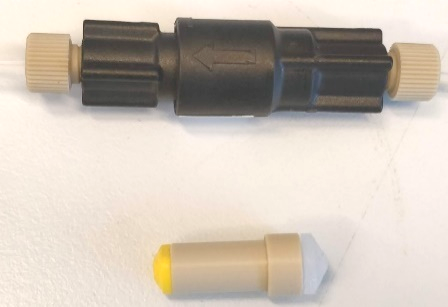


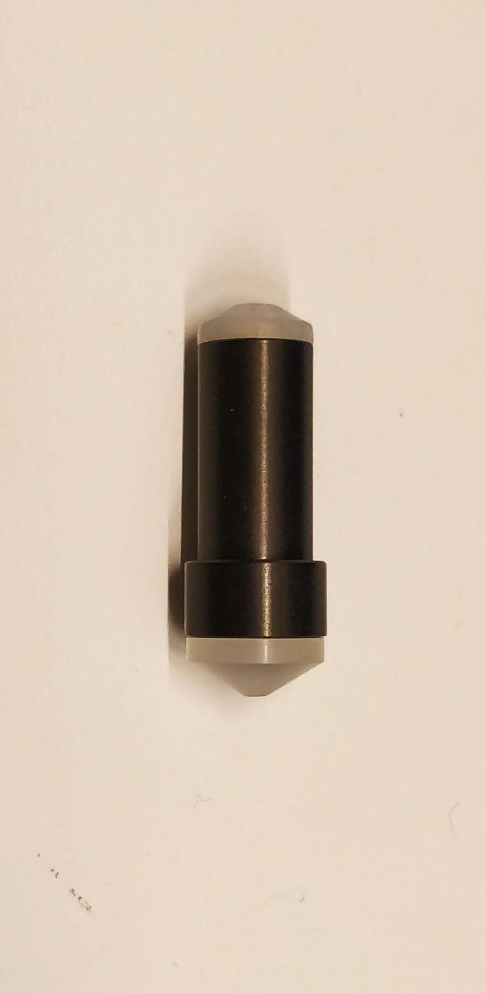


Syringe pump (Chemyx Fusion 200)

Gastight syringe (SGE Luer Lock 5)

HPLC pump (Shimadzu LC-20AD)

Mass Flow Controller (Bronkhorst EL-)

FLOW - Shut-Off

Valve (IDEX P-783) T-mixer (IDEX P-712)

BPR holder (IDEX P-789)

BPR cartridge

(IDEX P-789)

Check valve cartridge (IDEX CV-3000)

Figure S1: Flow equipment used for the photocatalytic reactions.

## Eagle Reactor

A Signify photochemical reactor is used, consisting of a base assembly with six 365 nm UV-A chip-on-board light modules.^2^ Each of these light source modules contain a fan and a heat sink to efficiently dissipate heat generated through the high power LEDs. Also the head cap assembly contains blowers to cool the interior of the reactor system, to reduce undesired thermal side-reactions. The LED modules and chamber cooling blowers are connected to a driver box, allowing to set the current of each of the LED modules individually, as well as the rotation speed of the cooling blowers. The six LED modules (365 nm, max. 144 W combined optical output power) are positioned in an hexagonal form around an aluminum cylinder support (80 mm height, 75 mm diameter), which has the reactor coil wrapped around (FEP capillary tubing: 1.6 mm OD, 0.8 mm ID, 11 mL volume or 0.5 mm ID, 2.8 mL volume).


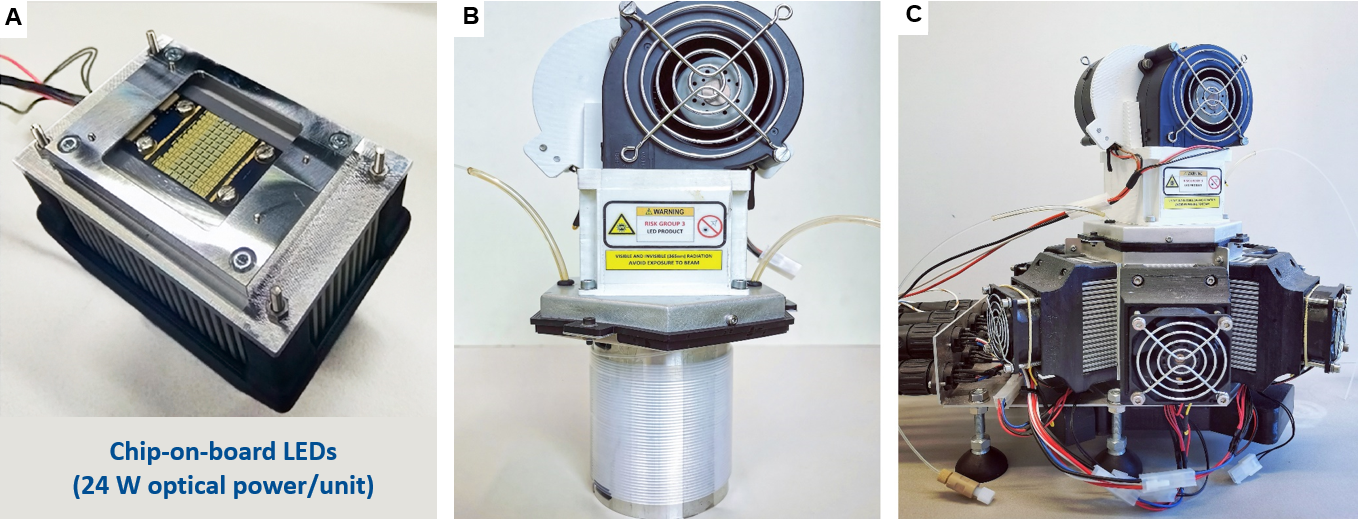


Figure S2 Signify Eagle Reactor with (A) six chip-on-board LED modules, (B) head assembly with reactor coil, and (C) complete assembly with fans, heat sinks and LED modules.


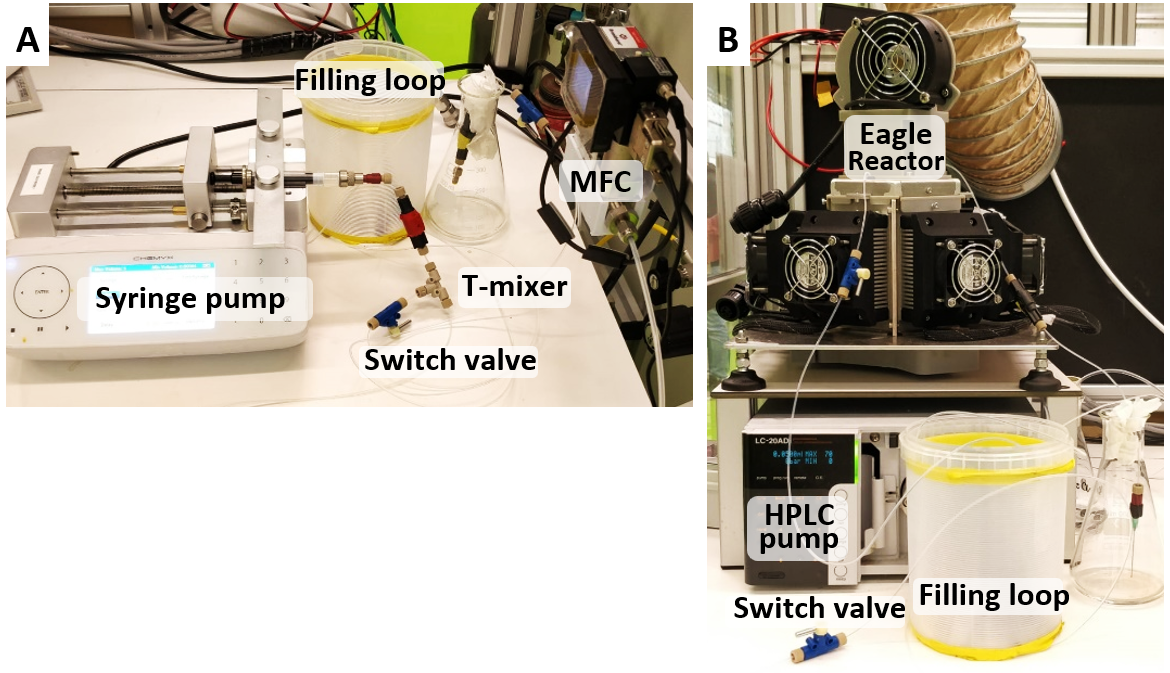


Figure S3: Overview of Setup for (A) loop filling of gas- and liquid, (B) photochemical reaction with Signify Eagle Reactor.

# General Procedure for the Optimization of gas-liquid Reactions in Flow

An elaborate description of the procedure for optimizing gas-liquid and gas-gas-liquid reactions in flow is described in the supporting information of our previous work on photocatalytic carbonylation of light and heavy hydrocarbons. ^3^ The relevant descriptions and calculations for this work are repeated here. For reactions above the maximum pressure of the liquid stream (syringe pump) or above the maximum pressure of the gas stream (pressure of the gas cylinder or reducer), a loop filling method is applied (Figure S4 A). With this method, the gas and liquid stream are first combined in a filling loop, then pressurized with a HPLC pump and finally injected into the reactor coil under the desired flow rate with the HPLC pump. (Figure S4 B)

Figure S2: Schematic representation of gas-liquid reactions performed with a loop filling method. (A) Loop filling of gas-liquid mixture, (B) Pressurizing the system and run the reaction using the correct flow rate. BPR: Back-Pressure Regulator.

The flow over the BPR only starts when the system has reached the design pressure of the BPR. The reaction is then performed through irradiation of the solution inside the reactor, and the reaction mixture is collected at the outlet.

# Reaction Optimization

The lactonization of Hexane-1-ol (**1**) using carbon monoxide (CO) was chosen as model reaction and an initial screening of reaction parameters was performed in the Signify Eagle Reactor (456 nm, 96 W output power) as described above.

## **Photocatalyst screening**

Table S1

| Photocatalyst | Yield of **1** | Yield of **2** |
| --- | --- | --- |
| [Ir{dF(CF_3_)ppy}_2_(dtbpy)]PF_6_ | 15% | 48% |
| [Ru(bpy)_3_]Cl_2_ | Traces | Traces |
| Eosin Y | nd | nd |
| [Ir{dF(CF_3_)ppy}_2_(bpy)]PF_6_ | 10% | 38% |
| Ir(ppy)_3_ | nd | 41% |
| 4CzIPN | 13% | 40% |
| 2,4,6-Triphenylpyrylium tetrafluoroborate | 12% | 41% |
| **9-Mesityl-10-methylacridinium tetrafluoroborate (PC1)** | **9%** | **58%** |
| PC2 | 11% | 39% |
| PC3 | 8% | 37% |
| PC4 | 20% | 25% |
| PC5 | 15% | 36% |

## Photocatalyst amount screening

Table S2

| Photocatalyst (x mol%) | Yield of **1** | Yield of **2** |
| --- | --- | --- |
| 1 mol% | 27% | 38% |
| **2 mol%** | **9%** | **58%** |
| 3 mol% | 10% | 43% |
| 5 mol% | 5% | 35% |

## Residence time screening

Table S3

| Residence Time [min] | Yield of **1** | Yield of **2** |
| --- | --- | --- |
| 45 min | 7% | 42% |
| **60 min** | **9%** | **58%** |
| 90 min | nd | 43% |

## Pressure screening

Table S4

| Pressure | Yield of **2** | Yield of **1** |
| --- | --- | --- |
| 69 bar | 55% | 11% |
| **80 bar** | **58%** | **9%** |
| 49 bar | 44% | 20% |
| 35 bar | 29% | 25% |

## Hypervalent iodine source screening

**Figure S5:** Screening of different hypervalent iodine sources. ^a^ Not amenable to flow. Insoluble hypervalent iodine sources.

## PIDA amount screening

Table S5

| Photocatalyst (x equiv.) | Yield of **1** | Yield of **2** |
| --- | --- | --- |
| 1 equiv. | 22% | 36% |
| **1.5 equiv.** | **9%** | **58%** |
| 1.8 equiv. | 10% | 51% |
| 2 equiv. | 8% | 52% |
| 2.5 equiv. | _^a^ | _^a^ |

^a^ Not amenable to flow. Insoluble hypervalent iodine sources.

## Additive screening

Table S6

| Additive | Yield of **2** | Yield of **1** |  |
| --- | --- | --- | --- |
| Tetra butyl ammonium chloride | 22% | 9% |  |
| Tetra butyl ammonium bromide | nd | 30% |  |
| Tetra butyl ammonium iodide | nd | 20% |  |
| Tetra butyl ammonium acetate | nd | 28% |  |
| Tetra butyl ammonium trifluoromethanesulphate | 43% | 5% |  |
| Tetra butyl ammonium tetrafluoroborate | 37% | 16% |  |
| Tetra butyl ammonium hexafluorophosphate | 42% | 10% |  |
| Tetra butyl ammonium hydrogen sulphate | 20% | 38% |  |
| I_2_ | nd | nd |  |
| **NFSI** | **58%** | **9%** |  |
| TBHP | nd | nd |  |
| DMSO | nd | nd |  |
| Selectfluor | 22% | 10% |  |

## NFSI amount screening

Table S7

| Photocatalyst (x equiv.) | Yield of **1** | Yield of **2** |
| --- | --- | --- |
| 1 equiv. | 12% | 44% |
| **0.5 equiv.** | **9%** | **58%** |
| 0.25 equiv. | 22% | 34% |

## Light intensity screening

Table S8

| Light Intensity | Yield of **2** | Yield of **1** |
| --- | --- | --- |
| 184 W | 45% | 16% |
| **96 W** | **58%** | **9%** |
| 36.8 W | 47% | 15% |

## Exploration of LMCT Photocatalysis

Table S9

| Photocatalyst | Yield of **2** | Yield of **1** |
| --- | --- | --- |
| FeCl_3_ | nd^a^ | nd^a^ |
| CeCl_3_ | nd^a^ | nd^a^ |
| 1 equiv. of FeCl_3_  without oxidant | nd^a^ | nd^a^ |

^a^ Decomposition of the starting material observed.

# General Procedures (GP)

## (GP1)

To a nitrogen-purged, screw-capped vial, fitted with a rubber septum and charged with Photocatalyst (2.4 mg, 6 µmol, 2 mol%), PIDA (145 mg, 0.45 mmol, 1.5 equiv.), NFSI (47 mg, 0.15 mmol, 0.5 equiv.), the alcohol (0.3 mmol, 1 equiv.) was solubilized in 3 mL CH_3_CN (0.1 M).

The stock solution is charged in a gastight syringe, positioned in a syringe pump and combined with a stream of CO gas (120 mL, 5.4 mmol, 18 equiv.) through a T-mixer into filling loop A, with a liquid flow rate of 0.1 mL·min^-1^ and a CO gas flow rate of 4 mL·min^-1^ (40 : 1 = gas : liquid).

A BPR of 2.8 bar is used during the loop filling.

Next, the filling loop is connected to the reactor, the system is pressurized to 80 bar using an HPLC pump and the reaction mixture is pumped over the Signify Eagle reactor (456 nm, 96 W output power, FEP capillary: 0.5 mm ID, 2.7 mL) at a flow rate of 0.045 mL·min^-1^, resulting in a residence time of 1 h. The obtained reaction mixture is collected into a vial and evaporated in vacuo. The residue is purified by column chromatography (*n*-pentane: Et_2_O).

## Scale-up

To a nitrogen-purged round bottom flask, fitted with a rubber septum and charged with Photocatalyst (16 mg, 39 µmol, 2 mol%), PIDA (966 mg, 3 mmol, 1.5 equiv.), NFSI (315 mg, 1 mmol, 0.5 equiv.), the alcohol (2 mmol, 1 equiv.) was solubilized in 20 mL CH_3_CN (0.1 M).

The stock solution is charged in a gastight syringe, positioned in a syringe pump and combined with a stream of CO gas (600 mL, 26.6 mmol, 13.3 equiv.) through a T-mixer into filling loop A, with a liquid flow rate of 0.15 mL·min^-1^ and a CO gas flow rate of 4 mL·min^-1^ (30 : 1 = gas : liquid).

A BPR of 2.8 bar is used during the loop filling.

Next, the filling loop is connected to the reactor, the system is pressurized to 80 bar using an HPLC pump and the reaction mixture is pumped over the Signify Eagle reactor (456 nm, 96 W output power, FEP capillary: 0.5 mm ID, 2.7 mL) at a flow rate of 0.045 mL·min^-1^, resulting in a residence time of 1 h. The obtained reaction mixture is collected into a vial and evaporated in vacuo. The residue is purified by column chromatography (*n*-pentane: Et_2_O) to afford the product **6** as a colourless oil (207 mg, 43% yield).

# Characterization data of synthesized compound

***3-ethyltetrahydro-2H-pyran-2-one* (2).** Prepared according to GP1 from hexan-1-ol (30.6 mg, 0.3 mmol, 1.0 equiv.). Purified via flash column chromatography on silica gel (from Pentane to Pentane:Et_2_O 5:1) to afford the product as a colorless oil (19.5 mg, 51% yield).

**^1^H NMR** (300 MHz, CDCl_3_) δ 4.36 – 4.19 (m, 2H), 2.38 (dtd, *J* = 10.8, 7.5, 5.2 Hz, 1H), 2.15 – 2.02 (m, 1H), 1.96 – 1.82 (m, 3H), 1.60 – 1.46 (m, 2H), 0.96 (t, *J* = 7.5 Hz, 3H).

**^13^C NMR** (101 MHz, CDCl_3_) δ 174.6, 68.5, 41.1, 24.3, 24.2, 22.1, 11.4.

The spectroscopic data are consistent with those reported previously^4^.

***3-methyltetrahydro-2H-pyran-2-one* (3).** Prepared according to GP1 from pentane-1-ol (26.4 mg, 0.3 mmol, 1.0 equiv.). Purified via flash column chromatography on silica gel (from Pentane to Pentane:Et_2_O 5:1) to afford the product as a colorless oil (17 mg, 49% yield).

**^1^H NMR** (300 MHz, CDCl_3_) δ 4.40 – 4.23 (m, 2H), 2.59 (dp, *J* = 11.1, 7.0 Hz, 1H), 2.17 – 2.03 (m, 1H), 1.99 – 1.83 (m, 2H), 1.55 (ddt, *J* = 13.2, 11.0, 7.3 Hz, 1H), 1.27 (d, *J* = 6.9 Hz, 3H).

**^13^C NMR** (101 MHz, CDCl_3_) δ 175.4, 68.6, 34.7, 27.2, 22.1, 16.7.

The spectroscopic data are consistent with those reported previously^4^.

***3-butyltetrahydro-2H-pyran-2-one* (4)** Prepared according to GP1 from octane-1-ol (39 mg, 0.3 mmol, 1.0 equiv.). Purified via flash column chromatography on silica gel (from Pentane to Pentane:Et_2_O 5:1) to afford the product as a colorless oil (26 mg, 55% yield).

**^1^H NMR** (300 MHz, CDCl_3_) δ 4.41 – 4.16 (m, 2H), 2.44 (dtd, *J* = 10.8, 7.6, 5.2 Hz, 1H), 2.18 – 2.03 (m, 1H), 1.88 (m, 3H), 1.58 – 1.43 (m, 2H), 1.33 (ddd, *J* = 7.1, 5.3, 4.1 Hz, 4H), 0.90 (td, *J* = 6.7, 3.4 Hz, 3H).

**^13^C NMR** (101 MHz, CDCl_3_) δ 174.90, 68.47, 39.70, 31.08, 29.14, 24.74, 22.75, 22.15, 14.07.

The spectroscopic data are consistent with those reported previously^4^.

***3-octyltetrahydro-2H-pyran-2-one* (5)** Prepared according to GP1 from dodecan-1-ol (56 mg, 0.3 mmol, 1.0 equiv.). Purified via flash column chromatography on silica gel (from Pentane to Pentane:Et_2_O 5:1) to afford the product as a colorless oil (36 mg, 56% yield).

**^1^H NMR** (400 MHz, CDCl_3_) δ 4.78 – 3.76 (m, 2H), 2.44 (dtd, *J* = 10.7, 7.6, 5.1 Hz, 1H), 2.09 (dq, *J* = 13.5, 6.8 Hz, 1H), 1.98 – 1.79 (m, 3H), 1.60 – 1.42 (m, 2H), 1.38 – 1.16 (m, 12H), 0.87 (t, *J* = 6.8 Hz, 3H).

**^13^C NMR** (75 MHz, CDCl_3_ δ 174.88, 68.47, 39.74, 31.99, 31.39, 29.69, 29.59, 29.39, 26.98, 24.75, 22.79, 22.16, 14.23.

**HRMS** (ESI) m/z calcd for C_13_H_24_O_2_ +: [M]+ 212.1771; found: 212.1773.

***3-decyltetrahydro-2H-pyran-2-one* (6)** Prepared according to GP1 from tetradecan-1-ol (64.2 mg, 0.3 mmol, 1.0 equiv.). Purified via flash column chromatography on silica gel (from Pentane to Pentane:Et_2_O 5:1) to afford the product as a colorless oil (39 mg, 54% yield).

**^1^H NMR** (300 MHz, CDCl_3_) δ 4.29 (td, *J* = 5.8, 2.5 Hz, 2H), 2.44 (dtd, *J* = 10.7, 7.5, 5.2 Hz, 1H), 2.09 (dq, *J* = 13.3, 6.9 Hz, 1H), 1.98 – 1.78 (m, 3H), 1.60 – 1.43 (m, 2H), 1.39 – 1.22 (m, 16H), 0.92 – 0.82 (m, 3H).

**^13^C NMR** (75 MHz, CDCl_3_) δ 174.9, 68.5, 39.7, 32.0, 31.4, 29.8, 29.7, 29.7, 29.6, 29.4, 27.0, 24.7, 22.8, 22.1, 14.2.

**HRMS** (ESI) m/z calcd for C_15_H_28_O_2_ +: [M]+ 240.2084; found: 240.2087.

***3-dodecyltetrahydro-2H-pyran-2-one* (7).** Prepared according to GP1, Modification 1 from hexadecan-1-ol (72.6 mg, 0.3 mmol, 1.0 equiv.). Purified via flash column chromatography on silica gel (from Pentane to Pentane:Et_2_O 5:1) to afford the product as a solid (39 mg, 35% yield).

**^1^H NMR** (300 MHz, CDCl_3_) δ 4.68 – 4.21 (m, 2H), 2.57 – 2.36 (m, 1H), 2.21 – 2.02 (m, 1H), 1.98 – 1.83 (m, 3H), 1.52 m, 2H), 1.28 (d, *J* = 4.7 Hz, 20H), 0.97 – 0.84 (m, 3H).

**^13^C NMR** (101 MHz, CDCl_3_) δ 174.7, 68.3, 39.6, 31.9, 31.3,29.7, 29.6, 29.6, 29.6, 29.5, 29.3, 26.9, 24.6, 22.7, 22.0, 14.1.

**HRMS** (ESI) m/z calcd for C_17_H_32_O_2_ + : [M]+ 268.2402; found: 268.2405.

***3-(3-phenylpropyl)tetrahydro-2H-pyran-2-one*** **(8).** Prepared according to GP1 from 7-phenylheptan-1-ol (57.6 mg, 0.3 mmol, 1.0 equiv.). Purified via flash column chromatography on silica gel (from Pentane to Pentane:Et_2_O 5:1) to afford the product as a colorless oil (31 mg, 47% yield).

**^1^H NMR** (400 MHz, CDCl_3_) δ 7.33 – 7.26 (m, 2H), 7.19 (dt, *J* = 6.0, 1.6 Hz, 3H), 4.52 – 4.10 (m, 2H), 2.64 (tt, *J* = 8.6, 4.4 Hz, 2H), 2.52-2.42 (m, 1H), 2.14-2.05 (m, 1H), 2.02 – 1.83 (m, 3H), 1.78 – 1.66 (m, 2H), 1.61 – 1.47 (m, 2H).

**^13^C NMR** (101 MHz, CDCl_3_) δ 174.5, 142.1, 128.4, 128.3, 125.8, 68.3, 39.5, 35.9, 31.0, 28.8, 24.6, 22.0.

**HRMS** (ESI) m/z calcd for C_14_H_18_O_2_ + : [M]+ 218.1301; found: 218.1301.

***4-(2-oxotetrahydro-2H-pyran-3-yl)butyl acetate* (9).** Prepared according to GP1 from 8-hydroxyoctyl acetate (57 mg, 0.3 mmol, 1.0 equiv.). Purified via flash column chromatography on silica gel (from Pentane to Pentane:Et_2_O 5:1) to afford the product as a colorless oil (24.5 mg, 38% yield).

**^1^H NMR** (300 MHz, CDCl_3_) δ 4.46 – 4.23 (m, 2H), 4.05 (q, *J* = 6.8 Hz, 2H), 2.68 – 2.33 (m, 1H), 2.16 – 2.05 (m, 1H), 2.04 (s, 3H), 1.95 – 1.83 (m, 2H), 1.72 – 1.59 (m, 2H), 1.59 – 1.38 (m, 3H).

**^13^C NMR** (101 MHz, CDCl_3_) δ 174.6, 171.4, 68.4, 64.4, 39.6, 31.0, 28.7, 24.7, 23.5, 22.1, 21.1.

**HRMS** (ESI) m/z calcd for C_11_H_18_NO_4_ +: [M]+ 214.1200; found: 214.1206.

***3-(4-chlorobutyl)tetrahydro-2H-pyran-2-one* (10).** Prepared according to GP1 from 8-chlorooctan-1-ol (49.2 mg, 0.3 mmol, 1.0 equiv.). Purified via flash column chromatography on silica gel (from Pentane to Pentane: Et_2_O 5:1) to afford the product as a colorless oil (21 mg, 37% yield).

**^1^H NMR** (300 MHz, CDCl_3_) δ 4.48 – 4.17 (m, 2H), 3.57 (t, *J* = 6.6 Hz, 2H), 2.48 (dtd, *J* = 10.9, 7.2, 5.3 Hz, 1H), 2.22 – 2.07 (m, 1H), 2.01 – 1.86 (m, 3H), 1.86 – 1.73 (m, 2H), 1.67 – 1.47 (m, 4H).

**^13^C NMR** (101 MHz, CDCl_3_) δ 174.5, 68.4, 44.9, 39.6, 32.6, 30.6, 24.7, 24.3, 22.1.

**HRMS** (ESI) m/z calcd for C_9_H_15_ClO_2_ + : [M]+190.0755; found:190.0756.

***4-(2-oxotetrahydro-2H-pyran-3-yl)butyl benzoate* (11).** Prepared according to GP1 from 8-hydroxyoctyl benzoate (75 mg, 0.3 mmol, 1.0 equiv.). Purified via flash column chromatography on silica gel (from Pentane to Pentane: Et_2_O 5:1) to afford the product as a colorless oil (50 mg, 60% yield).

**^1^H NMR** (400 MHz, CDCl_3_) δ 8.03 (d, *J* = 7.0 Hz, 2H), 7.59 – 7.51 (m, 1H), 7.43 (dd, *J* = 8.4, 7.0 Hz, 2H), 4.65 – 3.73 (m, 4H), 2.54 – 2.42 (m, 1H), 2.10 (dq, *J* = 13.5, 6.8 Hz, 1H), 2.00 – 1.92 (m, 1H), 1.92 – 1.86 (m, 2H), 1.84-1.74 (m, 2H), 1.61-1.49 (m, *J* = 14.2, 7.2, 2.6 Hz, 4H).

**^13^C NMR** (101 MHz, CDCl_3_) δ 174.6, 166.8, 133.0, 130.5, 129.6, 128.5, 68.4, 64.8, 39.6, 31.0, 28.9, 24.7, 23.6, 22.1.

**HRMS** (ESI) m/z calcd for C_16_H_20_O_4_ + : [M]+276.1362; found:276.1366.

***4-(2-oxotetrahydro-2H-pyran-3-yl) butyl 2-(4-isobutylphenyl) propanoate* (12).** Prepared according to GP1 from 8-hydroxyoctyl 2-(4-isobutylphenyl) propanoate (100.2 mg, 0.3 mmol, 1.0 equiv.). Purified via flash column chromatography on silica gel (from Pentane to Pentane: Et_2_O 5:1) to afford the product as a colorless oil (27 mg, 25% yield).

**^1^H NMR** (400 MHz, CDCl_3_) δ 7.19 (d, *J* = 7.9 Hz, 2H), 7.08 (d, *J* = 8.0 Hz, 2H), 4.41 – 4.20 (m, 2H), 4.07 (t, *J* = 6.5 Hz, 2H), 3.68 (q, *J* = 7.2 Hz, 1H), 2.44 (d, *J* = 7.2 Hz, 2H), 2.41 – 2.30 (m, 1H), 2.01 (dq, *J* = 13.4, 6.8 Hz, 1H), 1.94 – 1.76 (m, 4H), 1.69 – 1.53 (m, 2H), 1.48 (d, *J* = 7.1 Hz, 3H), δ 1.46 – 1.39 (m, 1H), 1.33 (p, *J* = 7.9 Hz, 2H), 0.89 (d, *J* = 6.6 Hz, 6H).

**^13^C NMR** (101 MHz, CDCl_3_) δ 174.9, 140.6, 138.0, 129.4, 127.3, 68.4, 64.5, 45.3, 45.2, 39.6, 30.9, 30.3, 28.6, 24.7, 23.3, 22.5, 22.1, 18.6.

**HRMS** (ESI) m/z calcd for C_22_H_32_O_4_ +: [M]+360.2295; found:360.2298.

***3-(hex-5-yn-1-yl)tetrahydro-2H-pyran-2-one* (13).** Prepared according to GP1 from dec-9-yn-1-ol (46.2 mg, 0.3 mmol, 1.0 equiv.). Purified via flash column chromatography on silica gel (from Pentane to Pentane: Et_2_O 5:1) to afford the product as a colorless oil (24 mg, 44% yield).

**^1^H NMR** (300 MHz, CDCl_3_) δ 4.72 – 4.19 (m, 2H), 2.55 – 2.36 (m, 1H), 2.21 (td, *J* = 6.7, 2.7 Hz, 2H), 2.17 – 2.05 (m, 1H), 1.97 – 1.80 (m, 4H), 1.58 (s, 1H), 1.57 – 1.42 (m, 5H).

**^13^C NMR** (101 MHz, CDCl_3_) δ 174.6, 84.4, 68.5, 68.4, 39.6, 30.8, 28.4, 26.1, 24.7, 22.2, 18.4.

**HRMS** (ESI) m/z calcd for C_11_H_16_O_2_ +: [M]+180.1145; found:180.1144.

***3-(hex-5-en-1-yl) tetrahydro-2H-pyran-2-one* (14).** Prepared according to GP1 from dec-9-en-1-ol (47 mg, 0.3 mmol, 1.0 equiv.). Purified via flash column chromatography on silica gel (from Pentane to Pentane:Et_2_O 5:1) to afford the product as a colorless oil (25 mg, 38% yield).

**^1^H NMR** (300 MHz, CDCl_3_) δ 5.77 (ddt, *J* = 16.9, 10.1, 6.7 Hz, 1H), 5.20 – 4.75 (m, 2H), 4.34 – 4.21 (m, 2H), 2.45 (dtd, *J* = 10.8, 7.4, 5.2 Hz, 1H), 2.15 – 1.99 (m, 3H), 1.95 – 1.78 (m, 3H), 1.61 – 1.45 (m, 2H), 1.44 – 1.18 (m, 4H).

**^13^C NMR** (75 MHz, CDCl_3_) δ 174.7, 138.8, 114.5, 68.4, 39.6, 33.6, 31.1, 28.8, 26.3, 24.6, 22.1.

**HRMS** (ESI) m/z calcd for C_11_H_18_O_2_ +: [M]+ 182.1301; found: 182.1300.

***4,4-dimethyltetrahydro-2H-pyran-2-one* (15).** Prepared according to GP1 from 3,3-dimethylbutan-1-ol (30.6 mg, 0.3 mmol, 1.0 equiv.). Purified via flash column chromatography on silica gel (from Pentane to Pentane:Et_2_O 5:1) to afford the product as a colorless oil (17 mg, 44% yield).

**^1^H NMR** (300 MHz, CDCl_3_) δ 4.38 – 4.34 (m, 2H), 2.32 (s, 2H), 1.69 (t, *J* = 6.1 Hz, 2H), 1.09 (s, 6H).

**^13^C NMR** (101 MHz, CDCl_3_) δ 171.7, 66.7, 44.4, 36.1, 29.9, 29.0.

**HRMS** (ESI) m/z calcd for C_7_H_12_O_2_ + : [M]+128.0832; found: 128.0835.

***3-methyl-5-propyltetrahydro-2H-pyran-2-one*** **(16).** Prepared according to GP1 from 2-propylpentan-1-ol (39 mg, 0.3 mmol, 1.0 equiv.). Purified via flash column chromatography on silica gel (from Pentane to Pentane:Et_2_O 5:1) to afford the product as a colorless oil (20.5 mg, 44% yield).

**^1^H NMR** (400 MHz, CDCl_3_) Obtained as a cis/trans-isomer mixture in a 1:1. δ 4.33 (ddd, *J* = 11.2, 4.9, 1.9 Hz, 1H), 4.25 (ddd, *J* = 11.0, 5.0, 0.9 Hz, 1H), 3.99 – 3.88 (m, 2H), 2.62 (ddt, *J* = 15.0, 9.5, 7.0 Hz, 1H), 2.52 (dt, *J* = 12.4, 6.8 Hz, 1H), 2.11 (dddd, *J* = 13.5, 6.8, 5.1, 2.0 Hz, 1H), 2.00 (ddddd, *J* = 15.1, 10.0, 6.4, 5.1, 2.3 Hz, 2H), 1.84 – 1.57 (m, 3H), 1.48 – 1.28 (m, 8H), 1.26 (d, *J* = 7.0 Hz, 3H), 1.22 (d, *J* = 6.8 Hz, 3H), 0.97 – 0.88 (m, 6H).

**^13^C NMR** (101 MHz, CDCl_3_) δ 176.5, 175.4, 74.0, 72.3, 35.7, 35.2, 35.1, 34.6, 33.7, 33.4, 32.7, 32.1, 21.1, 20.5, 20.3, 17.4, 17.0, 14.5.

The spectroscopic data are consistent with those reported previously^6^.

***3,5-dimethyltetrahydro-2H-pyran-2-one*** **(17).** Prepared according to GP1 from 2-methylpentan-1-ol (30.6 mg, 0.3 mmol, 1.0 equiv.). Purified via flash column chromatography on silica gel (from Pentane to Pentane:Et_2_O 5:1) to afford the product as a colorless oil (17.5 mg, 45% yield).

**^1^H NMR** (300 MHz, CDCl_3_) Obtained as a cis/trans-isomer mixture in a 1:1. δ 4.29 (dd, *J* = 4.7, 2.2 Hz, 1H), 4.22 (dd, *J* = 11.1, 5.0 Hz, 1H), 3.99 – 3.81 (m, 2H), 2.74 – 2.59 (m, 1H), 2.52 (dq, *J* = 12.5, 6.8 Hz, 1H), 2.26 – 1.99 (m, 3H), 1.81 – 1.63 (m, 2H), 1.24 (dd, *J* = 9.0, 6.9 Hz, 7H), 0.99 (dd, *J* = 8.2, 6.7 Hz, 6H).

**^13^C NMR** (101 MHz, CDCl_3_) δ 175.9, 174.7, 75.1, 73.3, 37.0, 35.5, 34.9, 32.4, 28.8, 26.8, 17.5, 17.1, 17.1, 16.9.

The spectroscopic data are consistent with those reported previously^5^.

***5-ethyltetrahydro-2H-pyran-2-one* (18).** Prepared according to GP1 from 2-ethylbutan-1-ol (30.6 mg, 0.3 mmol, 1.0 equiv.). Purified via flash column chromatography on silica gel (from Pentane to Pentane:Et_2_O 5:1) to afford the product as a colorless oil (18.5 mg, 44% yield).

**^1^H NMR** (300 MHz, CDCl_3_) δ 4.35 (ddd, *J* = 11.1, 4.6, 1.9 Hz, 1H), 3.96 (dd, *J* = 11.1, 9.7 Hz, 1H), 2.75 – 2.38 (m, 2H), 2.02 (dddd, *J* = 15.3, 9.4, 4.9, 1.9 Hz, 1H), 1.82 (ddtd, *J* = 11.9, 9.7, 4.9, 2.1 Hz, 1H), 1.59 – 1.42 (m, 2H), 1.40 – 1.29 (m, 2H), 0.96 (t, *J* = 7.4 Hz, 3H).

**^13^C NMR** (101 MHz, CDCl_3_) δ 171.8, 73.6, 34.6, 29.2, 25.3, 24.6, 11.4.

**HRMS** (ESI) m/z calcd for C_7_H_12_O_2_ + : [M]+ 128.0832; found: 128.0831.

***(4aR,6R,8S,10S,10aR)-octahydro-4a,8:6,10-dimethanocycloocta[c]pyran-1(5H)-one* (19).** Prepared according to GP1 from 2-((3r,5r,7r)-adamantan-1-yl)ethan-1-ol (54 mg, 0.3 mmol, 1.0 equiv.). Purified via flash column chromatography on silica gel (from Pentane to Pentane:Et_2_O 5:1) to afford the product as a colorless oil (32 mg, 52% yield).

**^1^H NMR** (400 MHz, CDCl_3_) δ 4.43 (ddd, *J* = 11.7, 9.2, 5.3 Hz, 1H), 4.34 (ddd, *J* = 11.6, 6.1, 5.0 Hz, 1H), 2.52 (p, *J* = 3.2 Hz, 1H), 2.42 (q, *J* = 2.1 Hz, 1H), 2.02 (dt, *J* = 9.9, 3.2 Hz, 2H), 1.97 – 1.89 (m, 1H), 1.88 – 1.79 (m, 2H), 1.77 (dt, *J* = 3.4, 1.5 Hz, 2H), 1.74 – 1.67 (m, 4H), 1.66 – 1.47 (m, 4H).

**^13^C NMR** (101 MHz, CDCl_3_) δ 173.3, 65.8, 51.0, 45.1, 39.5, 37.8, 36.6, 36.6, 32.4, 31.8, 28.3, 28.1, 27.9.

The spectroscopic data are consistent with those reported previously^6^.

***hexahydrocyclopenta[c]pyran-1(3H)-one* (20).** Prepared according to GP1 from 2-cyclopentylethan-1-ol (34.2 mg, 0.3 mmol, 1.0 equiv.). Purified via flash column chromatography on silica gel (from Pentane to Pentane:Et_2_O 5:1) to afford the product as a colorless oil (15 mg, 35% yield).

**^1^H NMR** (400 MHz, CDCl_3_) Obtained as a cis/trans-isomer mixture in a 1:6. δ 4.50 – 4.29 (m, 2H **trans**), 4.21 (td, *J* = 10.8, 2.4 Hz, 0.3H **cis**), 2.90 (dt, *J* = 10.3, 8.1 Hz, 0.3H **cis**), 2.50 (ddt, *J* = 17.5, 9.9, 7.6 Hz, 0.3H **cis**), 2.30 – 2.15 (m, 1.7H **cis+trans** ), 2.04 (tdd, *J* = 12.9, 9.1, 5.4 Hz, 3H **cis+trans**), 1.92 – 1.63 (m, 5H **cis+trans**), 1.61 – 1.49 (m, 1H **cis+trans**), 1.39 – 1.27 (m, 2H **cis+trans**).

**^13^C NMR** (101 MHz, CDCl_3_) δ 175.4 (**cis)**, 175.0 (**trans)**, 68.7 (**trans)**, 67.5 (**cis)**, 47.6 (**trans)**, 43.0(**cis)**, 40.3 (**trans)**, 36.4 (**cis)**, 33.8 (**cis)**, 31.8 (**trans)**, 30.0 (**cis)**, 29.3 (**cis)**, 28.2(**trans)**, 25.3 (**cis)**, 24.1(**trans)**, 22.2(**trans)**.

The spectroscopic data are consistent with those reported previously^6^.

***octahydro-1H-isochromen-1-one* (21).** Prepared according to GP1 from 2-cyclohexylethan-1-ol (38.4 mg, 0.3 mmol, 1.0 equiv.). Purified via flash column chromatography on silica gel (from Pentane to Pentane:Et_2_O 5:1) to afford the product as a colorless oil (19 mg, 41% yield).

**^1^H NMR** (300 MHz, CDCl_3_) Obtained as a cis/trans-isomer mixture in a 1:1. δ 4.37 (dtd, *J* = 10.7, 5.4, 2.6 Hz, 1H), 4.27 (dtd, *J* = 11.5, 8.9, 5.0 Hz, 1H), 2.70 (q, *J* = 4.8, 4.3 Hz, 0.5H **cis**), 2.30 – 2.11 (m, 1.5H **cis+trans**), 2.07 – 1.90 (m, 1H **cis+trans**), 1.90 – 1.79 (m, 1H **cis+trans**), 1.78 – 1.55 (m, 4H **cis+trans**), 1.55 – 1.43 (m, 1H **cis+trans**), 1.35 – 1.07 (m, 3H **cis+trans**).

**^13^C NMR** (75 MHz, CDCl_3_) δ 174.5, 173.9, 68.0, 66.9, 45.5, 40.4, 36.5, 33.7, 32.1, 31.4, 29.9, 28.4, 27.0, 26.0, 25.9, 25.5, 24.8, 22.7.

The spectroscopic data are consistent with those reported previously^6^.

***4-methyloctahydro-1H-isochromen-1-one* (22).** Prepared according to GP1 from 2-cyclohexylpropan-1-ol (42.6 mg, 0.3 mmol, 1.0 equiv.). Purified via flash column chromatography on silica gel (from Pentane to Pentane:Et_2_O 5:1) to afford the product as a colorless oil (22.6 mg, 45% yield).

**^1^H NMR** (300 MHz, CDCl_3_) mixture of 4 diastereomers (10:6:3:traces) δ 4.33 (dd, *J* = 11.3, 5.3 Hz, 1H), 4.10 (dd, *J* = 11.2, 4.0 Hz, 0.49H), 3.90 (dd, *J* = 11.3, 8.2 Hz, 0.88H), 2.66 (s, 0.13H), 2.52 – 2.41 (m, 0.13H), 2.37 – 2.10 (m, 1.38H) , 2.02 (dddd, *J* = 11.6, 6.9, 3.6, 2.0 Hz, 1.34H), 1.93 – 1.63 (m, 3H), 1.36 – 1.14 (m, 6H), 1.09 – 0.89 (m, 3.5H).

**^13^C NMR** (101 MHz, CDCl_3_) mixture of 4 diastereomers δ 174.0, 173.8, 173.2, 74.9, 73.7, 72.1, 45.2, 44.8, 43.5, 43.3, 40.9, 40.6, 40.2, 38.7, 34.3, 32.9, 31.3, 31.1, 31.0, 29.9, 29.8, 29.5, 27.6, 27.4, 27.2, 26.4, 26.1, 25.8, 25.5, 23.1, 22.8, 15.6, 13.8, 13.6, 11.4.

The spectroscopic data are consistent with those reported previously^6^.

***octahydrocyclohepta[c]pyran-1(3H)-one* (23).** Prepared according to GP1 from 2-cycloheptylethan-1-ol (42.6 mg, 0.3 mmol, 1.0 equiv.). Purified via flash column chromatography on silica gel (from Pentane to Pentane:Et_2_O 5:1) to afford the product as a colorless oil (18.6 mg, 37% yield).

**^1^H NMR** (300 MHz, CDCl_3_) Obtained as a cis/trans-isomer mixture in a 1:4. δ 4.55 – 4.08 (m, 2.6H **cis+trans**), 2.80 (ddd, *J* = 10.4, 8.8, 4.9 Hz, 0.36H **cis**), 2.40 (tdd, *J* = 9.9, 4.5, 2.3 Hz, 1H **trans**), 2.29 (td, *J* = 9.6, 3.8 Hz, 1.3H **cis+trans** ), 2.14 – 1.49 (m, 14H **cis+trans**), 1.48 – 1.21 (m, 2.3H **cis+trans**).

**^13^C NMR** (101 MHz, CDCl_3_) δ 175.6 (**cis)**, 175.3 (**trans)**, 67.4 (**trans)**, 66.9 (**cis)**, 46.9(**trans)**, 44.3 (**cis)**, 37.8 (**trans)**, 36.1(**trans)**, 36.0 (**cis)**, 32.7 (**cis)**, 31.6 (**trans)**, 31.3 (**cis)**, 30.8 (**cis)**, 30.2 (**trans)**, 28.7(**cis)**, 28.7 (**cis)**, 26.9(**trans)**, 26.6(**trans)**, 26.5(**cis)**, 26.3(**trans)**.

**HRMS** (ESI) m/z calcd for C_10_H_16_O_2_ + : [M]+ 168.1145; found: 168.1149.

***3-cyclohexyltetrahydro-2H-pyran-2-one*** (**24).** Prepared according to GP1 from 4-cyclohexylbutan-1-ol (46.8 mg, 0.3 mmol, 1.0 equiv.). Purified via flash column chromatography on silica gel (from Pentane to Pentane:Et_2_O 5:1) to afford the product as a colorless solid (27 mg, 37% yield).

**^1^H NMR** (300 MHz, CDCl_3_) δ 4.38 – 4.17 (m, 2H), 2.39 (m, 1H), 2.13 – 1.80 (m, 4H), 1.79 – 1.56 (m, 6H), 1.39 – 1.19 (m, 3H), 1.18 – 1.02 (m, 3H).

**^13^C NMR** (101 MHz, CDCl_3_) δ 173.8, 68.8, 45.8, 39.0, 30.8, 28.6, 26.7, 26.5, 26.4, 22.7, 21.0.

**HRMS** (ESI) m/z calcd for C_11_H_18_O_2_ + : [M]+182.1301; found: 182.1305.

# Mechanistic Investigation:

## Radical trapping with TEMPO:

To a nitrogen-purged, screw-capped vial, fitted with a rubber septum and charged with Photocatalyst (2.4 mg, 6 µmol, 2 mol%), PIDA (145 mg, 0.45 mmol, 1.5 equiv.), NFSI (47 mg, 0.15 mmol, 0.5 equiv.), TEMPO (2 equiv.) and the alcohol (0.3 mmol, 1 equiv.) was solubilized in 3 mL CH_3_CN (0.1 M).

The stock solution is charged in a gastight syringe and connected to the reactor. The reaction mixture was pumped through a Signify Eagle photoreactor (456 nm, 96 W output power) equipped with an FEP capillary (0.5 mm ID, 2.7 mL internal volume) at a flow rate of 0.045 mL·min⁻¹, corresponding to a residence time of 1 h. The collected reaction mixture was transferred to a vial and analysed by HRMS.

To a nitrogen-purged, screw-capped vial, fitted with a rubber septum and charged with Photocatalyst (2.4 mg, 6 µmol, 2 mol%), PIDA (145 mg, 0.45 mmol, 1.5 equiv.), NFSI (47 mg, 0.15 mmol, 0.5 equiv.), TEMPO (2 equiv.) and the alcohol (0.3 mmol, 1 equiv.) was solubilized in 3 mL CH_3_CN (0.1 M).

The stock solution is charged in a gastight syringe, positioned in a syringe pump and combined with a stream of CO gas (120 mL, 5.4 mmol, 18 equiv.) through a T-mixer into filling loop A, with a liquid flow rate of 0.1 mL·min^-1^ and a CO gas flow rate of 4 mL·min^-1^ (40 : 1 = gas : liquid).

A BPR of 2.8 bar is used during the loop filling.

Next, the filling loop is connected to the reactor, the system is pressurized to 80 bar using an HPLC pump and the reaction mixture is pumped over the Signify Eagle reactor (456 nm, 96 W output power, FEP capillary: 0.5 mm ID, 2.7 mL) at a flow rate of 0.045 mL·min^-1^, resulting in a residence time of 1 h. The collected reaction mixture was transferred to a vial and analysed by HRMS.

## Attempt to trap the acyl cation:

To a nitrogen-purged, screw-capped vial, fitted with a rubber septum and charged with Photocatalyst (2.4 mg, 6 µmol, 2 mol%), PIDA (145 mg, 0.45 mmol, 1.5 equiv.), NFSI (47 mg, 0.15 mmol, 0.5 equiv.), Corresponding nucleophile (0.9 mmol, 1 equiv.) and the alcohol (0.3 mmol, 1 equiv.) was solubilized in 3 mL CH_3_CN (0.1 M).

The stock solution is charged in a gastight syringe, positioned in a syringe pump and combined with a stream of CO gas (120 mL, 5.4 mmol, 18 equiv.) through a T-mixer into filling loop A, with a liquid flow rate of 0.1 mL·min^-1^ and a CO gas flow rate of 4 mL·min^-1^ (40 : 1 = gas : liquid).

A BPR of 2.8 bar is used during the loop filling.

Next, the filling loop is connected to the reactor, the system is pressurized to 80 bar using an HPLC pump and the reaction mixture is pumped over the Signify Eagle reactor (456 nm, 96 W output power, FEP capillary: 0.5 mm ID, 2.7 mL) at a flow rate of 0.045 mL·min^-1^, resulting in a residence time of 1 h. The collected reaction mixture was transferred to a vial and cby ^1^H-NMR and GC-FID analysis.

# References

1. Li, Jm.; Yong, Jp.; Huang, Fl. *et al.*, *Chem Nat Compd* **2012,** *48*, 103–105.
2. Wan, T.; Wen, Z.; Laudadio, G.; Capaldo, L.; Lammers, R.; Rincón, J. A.; García-Losada, P.; Mateos, C.; Frederick, M. O.; Broersma, R.; Noël, T., ACS Cent. Sci. **2022**, *8*, 51–56.
3. Raymenants, F.; Masson, T. M.; Sanjosé‐Orduna, J.; Noël, T. Efficient C(Sp^3^ )−H *Angew. Chemie Int. Ed.* **2023**, *62* (36).
4. Yang, X.-H.; Yue, H.-T.; Yu, N.; Li, Y.-P.; Xie, J.-H.; Zhou, Q.-L. *Chem. Sci.* **2017**, *8*, 1811– 1814.
5. Grünanger, C. U.; Breit, B. *Angew. Chemie Int. Ed.* **2010**, *49* (5), 967–970.
6. Tsunoi, S.; Ryu, I.; Okuda, T.; Tanaka, M.; Komatsu, M.; Sonoda, N., *J. Am. Chem. Soc.* **1998**, *120* (34), 8692–8701.

# NMR Spectra

^1^H NMR (300 MHz, CDCl_3_) of **2**

^13^C NMR (101 MHz, CDCl_3_) of **2**

^1^H NMR (300 MHz, CDCl_3_) of **3**

^13^C NMR (101 MHz, CDCl_3_) of **3**

^1^H NMR (300 MHz, CDCl_3_) of **4**

^13^C NMR (101 MHz, CDCl_3_) of **4**

^1^H NMR (400 MHz, CDCl_3_) of **5**

^13^C NMR (75 MHz, CDCl_3_) of **5**

^1^H NMR (300 MHz, CDCl_3_) of **6**

^13^C NMR (75 MHz, CDCl_3_) of **6**

^1^H NMR (300 MHz, CDCl_3_) of **7**

^13^C NMR (101 MHz, CDCl_3_) of **7**

^1^H NMR (400 MHz, CDCl_3_) of **8**

^13^C NMR (101 MHz, CDCl_3_) of **8**

^1^H NMR (300 MHz, CDCl_3_) of **9**

^13^C NMR (101 MHz, CDCl_3_) of **9**

^1^H NMR (300 MHz, CDCl_3_) of **10**

^13^C NMR (101 MHz, CDCl_3_) of **10**

^1^H NMR (400 MHz, CDCl_3_) of **11**

^13^C NMR (101 MHz, CDCl_3_) of **11**

^1^H NMR (400 MHz, CDCl_3_) of **12**

^13^C NMR (101 MHz, CDCl_3_) of 1**2**

^1^H NMR (300 MHz, CDCl_3_) of **13**

^13^C NMR (101 MHz, CDCl_3_) of **13**

^1^H NMR (400 MHz, CDCl_3_) of **14**

^13^C NMR (101 MHz, CDCl_3_) of **14**

^1^H NMR (300 MHz, CDCl_3_) of **15**

^13^C NMR (101 MHz, CDCl_3_) of **15**

^1^H NMR (400 MHz, CDCl_3_) of **16**

^13^C NMR (101 MHz, CDCl_3_) of **16**

^1^H NMR (300 MHz, CDCl_3_) of **17**

^13^C NMR (101 MHz, CDCl_3_) of **17**

^1^H NMR (300 MHz, CDCl_3_) of **18**

^13^C NMR (101 MHz, CDCl_3_) of **18**

^1^H NMR (400 MHz, CDCl_3_) of **19**

^13^C NMR (101 MHz, CDCl_3_) of **19**

^1^H NMR (400 MHz, CDCl_3_) of **20**

^13^C NMR (101 MHz, CDCl_3_) of **20**

^1^H NMR (300 MHz, CDCl_3_) of **21**

^13^C NMR (75 MHz, CDCl_3_) of **21**

^1^H NMR (300 MHz, CDCl_3_) of **22**

^13^C NMR (101 MHz, CDCl_3_) of **22**

^1^H NMR (300 MHz, CDCl_3_) of **23**

^13^C NMR (101 MHz, CDCl_3_) of **23**

^1^H NMR (400 MHz, CDCl_3_) of **24**

^13^C NMR (101 MHz, CDCl_3_) of **24**
